# Supplementary material for: Heat adaptation of phage T7 under an extended genetic code
Source: Virus Evol. 2021 Dec 1;7(2):veab100. doi: 10.1093/ve/veab100 (PMC8923235; doi:10.1093/ve/veab100)
Supplement: veab100_Supp [file veab100_supp.zip › Sequencing_Analysis.zip!/Sequencing_Analysis/S1/evidence/RA_24.html]

BRESEQ :: Evidence


| Read alignment evidence... | | | | | | | | | | | |
| --- | --- | --- | --- | --- | --- | --- | --- | --- | --- | --- | --- |
|  | seq id | position | | ref | new | freq | score (cons/poly) | reads | annotation | genes | product |
| \* | NC\_001604.1 | 26,148 | 0 | A | G | 79.4% | 6777.8 / inf | 4179 | intergenic (–/–) | *–/–* | –/– |
| Reads supporting (aligned to +/- strand):  **ref** base A (420/374);  **new** base G (1500/1557);  **total** (2066/2113) | | | | | | | | | | | |
| Fisher's exact test for biased strand distribution *p*-value = 5.59e-02 | | | | | | | | | | | |
| Kolmogorov-Smirnov test that lower quality scores support variant *p*-value = 9.00e-01 | | | | | | | | | | | |

Only 100 of 4239 total aligned reads displayed.  

`CACCAACCCTTGGCCTTCTTTTGTTGGTTCAAGTATTAACGATGTGTTCTTCTTCCGTAACCGCTTAGGATTCCTTAGTGGGGAGAACATCATATTGAGTCGTACAGCCAAATACTTCAACTTCTACCCTGCGTCCATTGCGAACCTTAGTGATGACGACCCTATAGACGTAGCTGTGAGTACCAACCGAATAGCAATCCTTAAGTACGCCGTTCCGTTCTCAGAAGAGTTACTCATCTGGTCCGATGAAGCACAATTCGTCCTGACTGCCTCGGGTACTCTCACATCTAAGTCGGTTGAGTTGAACCTAACGACCCAGTTTGACGTACAGGACCGAGCGAGACCTTTTGGGATTGGGCGTAATGTCTACTTTGCTAGTCCGAGGTCCAGCTTCACGTCCATCCACAGGTACTACGCTGTGCAGGATGTCAGTTCCGTTAAGAATGCTGAGGACATTACATCACACGTTCCTAACTACATCCCTAATGGTGTGTTCAGTATTTGCGGAAGTGGTACGGAAAACTTCTGTTCGGTACTATCTCACGGGGACCCTAGTAAAATCTTCATGTACAAATTCCTGTACCTGAACGAAGAGTTAA  >  NC_001604.1/25849‑26447`  

                                                                                                                                                                                                                                                                                                           `|`                                                                                                                                                                                                                                                                                                             
      `cccTTGGCCTTCTTTTGTTGGTTCAAGTATTAAAGATGTGTTCTTCTTCCGTAACCGCTTAGGATTCCTTAGTGGGGAGAACATCATATTGAGTCGTACAGCCAAATACTTCAACTTCTACCCTGCGTCCATTGCGAACCTTAGTGATGACGACCCTATAGACGTAGCTGTGAGTACCAACCGAATAGCAATCCTTAAGTACGCCGTTCCGTTCTCAGAAGAGTTACTCATCTGGTCCGATGAAGCACAATTCGTCCTGACTGCCCAGGGTACTCTCACATCTAAGTCGGTTGGGTTGAAc                                                                                                                                                                                                                                                                                                      >  1:207608/1‑301 (MQ=255)`  
      `ataagagacagcttTTGTTGGTTCAAGTATTAACGATGTGTTCTTCTTCCGTAACCGCTTAGGATTCCTTAGTGGGGAGAACATCATATTGAGTCTTACAGCCAAATACTTCAACTTCTACCCTGCGTCCATTGCGAACCTTAGTGATGACGACCCTATAGACGTAGCTGTGAGTACCAACCGAATAGCAATCCTTAAGTACGCCGTTCCGTTCTCAGAAGAGTTACTCATCTGGTCCGATGAAGCACAATTCGTCCTGACTGCCTCGGGTACTCTCACATCTAAGTCGGTTGGGTTGAAc                                                                                                                                                                                                                                                                                                      <  1:354662/290‑1 (MQ=255)`  
               `ttcttTTGTTGGTTCAATTATTAACGATGTGTTCTTCTTCCGTAACCGCTTAGGATTCCTTAGTGGGGAGAACATCATATTGAGTCGTACAGCCAAATACTTCAACTTCTACCCTGCGTCCATTGCGAACCTTAGTGATGACGACCCTATAGACGTAGCTGTGAGTACCAACCGAATAGCAATCCTTAAGTACACCGTTCCGTTCTCAGAAGAGTTACTCATCTGGTCCGATGAAGCACAATTCGTCCTGACTGCCTCGGGTACTCTCACATCTAAGTCGGTTGGGTTGAACCTAACGAcc                                                                                                                                                                                                                                                                                             <  1:592181/301‑1 (MQ=255)`  
                          `gTTCAAGTATTAACGATGTGTTCTTCTTCCGTAACCGCTTAGGATTCCTTAGTGGGGAGAACATCATATTGAGTCGTACAGCCAAATACTTAAACTTCTACCCTGCGTCCATTGCGAACCTTAGTGATGACGACCCTATAGACGTAGCTGTGAGTACCAACCGAATAGCAATCCTTAAGTACGCCGTTCCGTTCTCAGAAGAGTTACTCATCTGGTCCGATGAAGCACAATTCGTCCTGACTGCCTCGGGTACTCTCACATCTAAGTCGGTTGGGTTGAACCTAACGACCCAGTTTGACGt                                                                                                                                                                                                                                                                                  >  1:471320/1‑301 (MQ=255)`  
                                `gTATTAACGATGTGTTCTTCTTCCGTAACCGCTTAGGATTCCTTAGTTGGGAGAACATCATATTGAGTCGTACAGCCAAATACTTCAACTTCTACCCTGCGTCCATTGCGAACCTTAGTGATGACGACCCTATAGACGTAGCTGTGAGTACCAACCGAATAGCAATCCTTAAGTACGCCGTTCCGTTCTCAGAAGAGTTACTCATCTGGTCCGATGAAGCACAATTCGTCCTGACTGCCTCGGGTACTCTCACATCTAAGTCGGTTGGGTTGAACCTAACGACCCAGTTTGACGTACAGGa                                                                                                                                                                                                                                                                            >  1:263975/1‑301 (MQ=255)`  
                                `gTATTAACGATGTGTTCTTCTTCCGTAACCGCTTAGGATTCCTTAGTGGGGAGAACATCATATTGAGTCGTACAGCCAAATACTTCAATTTCAACCCTGCGTCCATTGCGAACCTTAGTGATGACGAACCTCTAGACGTAGCTGTGAGTACCAACCGAATAGCAATCCTTAAGTTCGCCGTTCCGTTCCCAGAAGAGTTACTTATCTGGTGCGATGAAGCACAATTCGTCCTGACTGCCGCGGGTACTCTCACATCTAAGTCGGTTGGGTTGAACCTAACGACCCAGTTTGACGtctgttt                                                                                                                                                                                                                                                                            >  1:412135/1‑295 (MQ=255)`  
                                `gTATTAACGATGTGTTCTTCTTCCGTAACCGCTTAGGATTCCTTAGTGGGGAGAACATCATATTGAGTCGTACAGCCAAATACTTCAACTTCTACCCTGCGTCCATTGCGAACCTTAGTGATGACGACCCTATAGACGTAGCTGTGAGTACCAACCGAATAGCAATCCTTAAGTACGCCGTTCCGTTCTCAGAAGAGTTACTCATCTGGTCCGATGAAGTACAATTCGTCCTGACTGCCTCGGGTACTCTCACATCTAAGTCGGTTGGGTTGAACCTAACGACCCAGTTTGACGTACAgct                                                                                                                                                                                                                                                                            >  1:248392/1‑299 (MQ=255)`  
                                `gTATTAACGATGTGTTCTTCTTCCGTAACCGCTTAGGATTCCTTAGTGGGGAGAACATCATATTGAGTCGTACAGCCAAATACTTCAACTTCTACCCTGCGTCCATTGCGAACCTTAGTGATGACGACCCTATAGACGTAGCTGTGAGTACCAACCGAATAGCAATCCTTAAGTACGCCGTTCCGTTCTCAGAAGAGTTACTCATCTGGTCCGATGAAGCACAATTCGTCCTGACTGCCTCGGGTACTCTCACATCTAATTCGGTTGGTTTGAACCTAACGACCCAGTTTGACGTACAGGa                                                                                                                                                                                                                                                                            >  1:217020/1‑301 (MQ=255)`  
                                `gTATTAACGATGTGTTCTTCTTCCGTAACCGCTTAGGATTCCTTAGTGGGGAGAACATCATATTGAGTCGTACAGCCAAATACTTCAACTTCTACCCTGCGTCCATTGCGAACCTTAGTGATGACGACCCTATAGACGTAGCTGTGAGTACCAACCGAATAGCAATCCTTAAGTACGCCGTTCCGTTCTCAGAAGAGTTACTCATCTGGTCCGATGAAGCACAATTCGTCCTGACTGCCTCGGGTACTCTCACATCTAAGTCGGTTGGGTTGAACCTAACGACCCAGTTTGACGTACAGGa                                                                                                                                                                                                                                                                            >  1:548282/1‑301 (MQ=255)`  
                                 `tATTAACGATGTGTTCTTCTTCCGGAACCGCTTAGGATTCCTTAGTGGGGAGAACATCATATTGAGTCGTACAGCCAAATACTTCAACTTCTACCCTGCGTCCATTGCGAACCTTAGTGATGACGACCCTATAGACGTAGCTGTGAGTACCACCCGAATAGCAATCCTTAAGTACGCCGTTCCGTTCTCAGAAGAGTTACTCATCTGGTCCGATGAAGCACAATTCGTCCTGACTGCCTCGGGTACGCTCACATCTAAGTCGGTTGGGTTGAACCTAACGACCCAGTTTGACGTACAGGAc                                                                                                                                                                                                                                                                           <  1:487771/301‑1 (MQ=255)`  
                                  `aTTAACGATGTGTTCCTCTTCCGTAACAGCTTAGGATTCCTTAGTGGGGAGAACATCATATTGAGTCGTACAGCCAAATACTTCAACTTCTACCCTGCGTCCATTGCGAACCTTAGTGATGACGACCCTATAGACGTAGCTGTTAGTACCAACCGAATCGCAATCCTTAAGTACGCCGTTCCGTTCTCAGAAGAGTTACTCATCTGGTCCGATGAAGCACAATTCGTCCTGACTGCCTAGGGTACTCTCACATCTAAGTCGGTTGGGTTGAACCTAACGAACCAGTTTGACGTACAGGGcc                                                                                                                                                                                                                                                                          >  1:640718/1‑301 (MQ=255)`  
                                      `aCGATGTGTTCTTCTTCCGTAACCGCTTAGGATTCCTTAGTGGGGAGAACATCATATTGAGTCGTACAGCCAAATACTTCAACTTCTACCATGCGTCCATTGCGAACCTTAGTGATGACGACCCTATAGACGTAGCTGTGAGTACCAACCGAATAGCAATCCTTAAGTACGCCGTTCCGTTCTCAGAAGAGTTACTCATCTGGTCCGATGAAGCACAATTCGTCCTGACTGCCTCGGGTACTCTCACATCTAAGTCGGTTGGGTTGAACCTAACGACCCAGTTTGACGTACAGGACcgagc                                                                                                                                                                                                                                                                      <  1:536106/301‑1 (MQ=255)`  
                                       `caaTGTGTTCTTCTTCCGGAACCGCTTAGGGTTCCTTAGTGGGGAGAACATCATATTGAGTCGTACAGCCAAATACTTCAACTTCTACCCTGCGTCCATTGCGAACCATAGTGATGACGACCCTATAGACGTAGCTGTGAGTACCAACCGAATAGCAATCCTTAAGTACGCCGTTCCGTTCTCAGAAGAGTTACTCATCTGGTCCGATGAAGCACAATTCGTCCTGACTGCCTCGGGTACTCTCACATCTAAGTCGGTTGGGTTGAACCTAACGACCCAGTTTGACGTACAGGACcgagcg                                                                                                                                                                                                                                                                     <  1:322669/299‑1 (MQ=255)`  
                                                    `ttcCGTAACCGCTTAGGATTCCTTAGTGGGGAGAACATCATATTGAGTCGTACAGCCAAATACTTCAACTTCTACCCTGCGTCCATTGCGAACCTTAGTGATGACGACCCTATAGACGTAGCTGTTAGTACCAACCGAATAGCAATCCTTAAGTACGCCGTTCCGTTCTCAGAATAGTTACTCATCTGGTCCGATGAAGCACAATTCGTCCTGACTGCCTCGGGTACTCTCACATCTAAGTCGGTTGGGTTGAACCTAACGACCCAGTTTGACGTACAGGACCGAGCGAGACCTTTTGGGa                                                                                                                                                                                                                                                        <  1:112625/301‑1 (MQ=255)`  
                                                           `aCCGCTTAGGATTCCTTAGTGGGGAGAACATCATATTGAGTCGTACAGCCAAATACTTCAACTTCTACCCTGCGTCCATTGCGAACCTTAGTGATGACGACCCTATAGACGTAGCTGTGAGTACCAACCGAATAGCAATCCTTAAGTACGCCGTTCCGTTCTCAGAAGAGTTACTCATCTGGTCCGATGAAGCACAATTCGTCCTGACTGCCTCGGGTACTCTCACATCTAAGTCGGTTGGGTTGAACCTAACGACCCAGTTTGACGTACAGGACCGAGCGAGACCTTTTGGGATTGGGCg                                                                                                                                                                                                                                                 <  1:553074/301‑1 (MQ=255)`  
                                                                `ttAGGATTCCTTAGTGGGGAGAACATCATATTGAGTCGTACAGCCAAATACTTCAACTTCTACCCTGCGTCCATTGCGAACCTTAGTGATGACGACCCTATAGACGTAGCTGTGAGTACCAACCGAATAGCAATCCTTAAGTACGCCGTTCCGTTCTCAGAAGAGTTACTCATCTGGTCCGATGAAGCACAATTCGTCCTGACTGCCTCGGGTACTCTCACATCTAAGTCGGTTGGGTTGAACCTAACGACCCAGTTTGACGTACAGGACCGAGCGAGACCTTTTGGGATTGGGCGTAATg                                                                                                                                                                                                                                            <  1:26858/301‑1 (MQ=255)`  
                                                                        `ccTTAGTGGGGAGAACATCATATTGAGTCGTACAGCCAAATACTTCAACTTCTACCCTTCGTCCATTGCGAACCTTAGTGATGACGACCCTATAGACGTAGCTGTGAGTACCAACCGAATAGCAATCCTTAAGTACGCCGTTCCGTTCTCAGAAGAGTTACTAATCTGGTCCGATGAAGCACAATTCGTCCTGACTGCCTCGGGTACTATAACATCTAAGTCGGTTGGGTTGAACCTAACGACCCAGTTTGACGTACAGGACCGAGCTAGACCTTTTGGGATTGGGCGTAATGTCTACttt                                                                                                                                                                                                                                    >  1:187369/1‑301 (MQ=255)`  
                                                                        `ccTTAGTGGGGAGAACATCATATTGAGTCGTACAGCCAAATACTTCAACTTCTACCCTGCGTCCATTGCGAACCTTAGTGCTGACGACCCTATAGACGTATCTGTTAGTACCAACCGAATAGCAATCCTTAAGTACGCCGTTCCGTTCACCGAAGAGTTACTCACCTGCTCCGATGAAGCCCAATTCGTCCTGACTGCCTCGGGTACTCTCACATCTCAGTCGGTCGGGTTGAACCTAACGACCCAGTTTGTCGTACAGGACCGAGCGAGACCTTTTGGCTTTGGGCGTAATGTCTAtttt                                                                                                                                                                                                                                    >  1:282522/1‑301 (MQ=255)`  
                                                                          `ttAGTGGGGAGAACATCATATTGAGTCGTACAGCCAAATACTTCAACTTCTACCCTGCGTCCATTGCGAACCTTAGTGATGACGACCCTATAGACGTAGCTGTGAGTACCAACCGAATAGCAATCCTTAAGTACGCCGTTCCGTTCTCAGAAGAGTTACTCATCTGGTCCGATGAAGCACAATTCGTCCTGACTGCCTCGGGTACTCTCACATCTAAGTCGGTTGCGTTGAACCTAACGACCCAGTTTGACGTACAGGACCGAGCGAGACCTTTTGGGATTGGGCGTAATGTCTACTTTGc                                                                                                                                                                                                                                  <  1:160762/301‑1 (MQ=255)`  
                                                                          `caggTGGGGAGAACATCATATTCAGTCGTACAGCCAAACACTTCAACTTCTACCCTGCGTCCATTGCGAACCTTAGTGATGACGACCCTATAGACGTAGCTGTGAGTACCAACCGAAGAGCAATCCTTAAGTACGCCGGTCCGTTCTCAGAAGAGTTACTCATCTGGTCCGATGAAGCACAATTCGTCCTGACTGCCTCGGGTACTCTCACATCTAAGTCGGTTGGGTTGAACCTAACGACCCAGTTTGACGTACAGGACCGAGCGAGACCTTTTGGGATTGGGCGTAATGTCTACTTTGc                                                                                                                                                                                                                                  <  1:201034/298‑1 (MQ=255)`  
                                                                               `ggggAGAACATCATATTGATTCGTACAGCCAAATAATTCAACTTCTACCCTGCGTCCATTGCGAACCTTAGTGATGACGACCAAATAGACGTAGATGTGAGTACCAACCGAATAGACATCCTTAAGTATGCCGTTCCGTTCTAAGAAGAGTTACTCATATGGTCCGATGAAGAACAATTCGTCATGATAGCATCGGGTACTCTCACATCTAAGTCGGTAGGGTTGAACCTAACGACCCAGTTTGACGTAAAGGACCGAGCGAGACAGCTGGGGATTGGGCGTaagagctacctttgtagtg                                                                                                                                                                                                                             >  1:362762/1‑284 (MQ=255)`  
                                                                                    `gagaaagatatGGAGTCGTCCAGCCAAATACTTCAACTGCTACCCTGCGTCCATGGCGAACCTTAGTGATGACGACCCAATAGACGTAGCTGTGAGTACCAACCGAATAGCAATCCTTAAGTACGCCGTTCCGTTCTCAGAAGAGTTACTCATCTGGTCCGATGAAGCACAATTCGTCCTGACTGCCTCGGGTACTCTCACATCTAAGTCGGTTGGGTTGAACCTAACGACCCAGTTTGACGTACAGGACCGAGCGAGACCTTTTGGGATTGGGCGTAATGTCTACTTTGCTAGTCCGAgg                                                                                                                                                                                                                        <  1:39263/294‑1 (MQ=255)`  
                                                                                    `gaACATCATATTGAGTCGTACAGCCAAATACTTCAACTTCTACCCTGCGTCCATTGCGAACCTTAGTGATGACGACCCTATAGACGTAGCTGTGAGTACCAACCGAATAGCAATCCTTAAGTACGCCGTTCCGTTCTCAGAAGAGTTACTCATCTGGTCCGATGAAGCACAATTCGTCCTGACTGCCTCGGGTACTCTCACATCTAAGTCGGTTGGGTTGAACCTAACGACCCAGTTTGACGTACAGGACCGAGCGAGACCTTTTGGGATTGGGCGTAATGTCTACTTTGCTAGTCCgggg                                                                                                                                                                                                                        >  1:399622/1‑301 (MQ=255)`  
                                                                                    `gaACATCATATTGAGTCGTACAGCCAAATACTTCAACTTCTACCCTGCGTCCATTGCGAACCTTAGTGATGACGACCCTATAGACGTAGCTGTGAGTACCAACCGAATAGCAATCCTTAAGTACGCCGTTCCGTTCTCAGAAGAGTTACTCATCTGGTCCGATGAAGCACAATTCGTCCTGACTGCCTCGGGTACTCTCACATCTAAGTCGGTTGGGTTGAACCTAACGACCCAGTTTGACGTACAGGACCGAGCGAGACCTTTTGGGATTGGGCGTAATGTCTACTTTGCTAGTCCGAgg                                                                                                                                                                                                                        >  1:29637/1‑301 (MQ=255)`  
                                                                                      `aCATCATATTGAGTCGTACAGCCAAATACTTCAACTTCTACCCTGCGTCCATTGCGAACCTTAGTGATGACGACCCTATAGACGTAGCTGTGAGTACCAACCGAATAGCAATCCTTAAGTACGCCGTTCCGTTCTCAGAAGAGTTACTCATCTGGTCCGCTGAAGCACAATTCGTCCTGACTGCCTCGGGTACTCTCACATCTAAGTCGGTTGCGTTGAACCTAACGACCCAGTTTGACGTCCAGGACCGAGCGAGACCTTTTGGGATTCGGCGTAATGTCTACTTTTCTATTCCCAGGTc                                                                                                                                                                                                                      >  1:118371/1‑301 (MQ=255)`  
                                                                                      `aCATCATATTGAGTCGTACAGCCAAATACTTCAACTTCTACCCTGCGTCCATTGCGAACCTTAGTGATGACGACCCTATAGACGTAGCTGTGAGTACCAACCGAATAGCAATCCTTAAGTACGCCGTTCCGTTCTCAGAAGAGTTACTCATCTGGTCCGATGAAGCACAATTCGTCCTGACTGCCTCGGGTACTCTCACATCTAAGTCGGTTGGGTTGAACCTAACGACCCAGTTTGACGTACAGGACCGAGCGAGACCTTTTGGGATTGGGCGTAATGTCTACTTTGCTAGTCCGAGGTc                                                                                                                                                                                                                      >  1:6920/1‑301 (MQ=255)`  
                                                                                      `aCATCATATTGAGTCGTACAGCCAAATACTTCAACTTCTACCCTGCGTCCATTGCGAACCTTAGTGATGACGACCCTATAGACGTAGCTGTGAGTACCAACCGAATAGCAATCCTTAAGTACGCCGTTCCGTTCTCAGAAGAGTTACTCATCTGGTCCGATGAAGCACAATTCGTCCTGACTGCCTAGGGTACTCTCACAGCCAAGTCGGTTGGGTTGAACCTAACGACCCAGTTTGACGTACAGGACCGAGCGAGACCTGTTGTGAATGGGCGTAATGTCTATGTTGCTAGTCCGAGGTc                                                                                                                                                                                                                      >  1:661292/1‑301 (MQ=255)`  
                                                                                       `catcatATTGAGTCGTCCAGCCAAATACTTCAACTTCTACCCTGCGTCCATTGCGAACCTTAGTGATGACGACCCTATAGACGTAGCTGTGAGTACCAACCGAATAGCAATCCTTAAGTACGCCGTTCCGTTCTCAGAAGAGTTACTCATCTGGTCCGATGAAGCACAATTCGTCCTGACTGCCTCGGGTACTCTCACATCTAAGTCGGTTGAGTTGAACCTAACGACCCAGTTTGACGTACAGGACCGAGCGAGACCTTTTGGGATTGGGCGTAATGTCTAcctgtctcttatacacatc                                                                                                                                                                                                                     >  1:598970/1‑283 (MQ=255)`  
                                                                                       `aatcatATTGAGTCGTACAGCCAAATACTTCAACTTCTACCCTGCGTCCATTGCGAACCTTAGTGATGACGACCCTATAGACGTAGCTGTGAGTACCAACCGAATAGCAATCCTTAAGTACGCCGTTCCGTTCTCAGAAGAGTTACTCATCTGGTCCGATGAAGCACAATTCGTCCTGACTGCCTCGGGTACTCTCACATCTAAGTCGGTTGGGTTGAACCTAACGACCCAGTTTGACGTACAGGACCGAGCGAGACCTTTTGGGATTGGGCGTAATGTCTACTTTGCTAGTCCGAGGTcc                                                                                                                                                                                                                     <  1:573574/300‑1 (MQ=255)`  
                                                                                             `atTGAGTCGTACAGCCAAATACTTCAACTTCTACCCTGCGTCCATTGCGAACCTTAGTGATGACGACCCTATAGACGTAGCTGTGAGTACCAACCGAATAGCAATCCTTAAGTACGCCGTTCCGTTCTCAGAAGAGTTACTCATCTGGTCCGATGAAGCACAATTCGTCCTGACTGCCTCGGGTACTCTCACATCTAAGTCGGTTGGGTTGAACCTAACGACCCAGTTTGACGTACAGGACCGAGCGAGACCTTTTGGGATTGGGCGTAATGTCTACTTTGCTAGTCCGAGGTCCAGCTTc                                                                                                                                                                                                               <  1:342073/301‑1 (MQ=255)`  
                                                                                                         `aGCCAAATACTTCAACTTCTACCCTGCTTCCATTGCGAACCTTAGTTATTACGACCCTATAGACGTAGCTGTGAGTACCAACCGAATAGCAATCCTTAAGTACCCCGTTCCGTTCTCAGAAGAGTTACTCATCTGGTCCGATGAAGCACAATTCGTCCTGACTGCCTCGGGTACTCTCACATCTAAGTCGGTTGCGTTGAACCTAACTACCCAGTTTGACGTACAGGACCGATCGAGACCTTTTGGGATTGTGCGTAATGTCTACTTTGCTAGTCCTAGGTCCAGCTTCACGTCCATCcac                                                                                                                                                                                                   <  1:348282/301‑1 (MQ=255)`  
                                                                                                          `gCCAAATACTTCAACTTCTACCCTGCGTCCATTGCGAACCTTAGTGATGACGACCCTATAGACGTAGCTGTGAGTACCAACCGAATAGCAATCCTTAAGTACGCCGTTCCGTTCTCAGAAGAGTTACTCATCTGGTCCGATGAAGCACAATTCGTCCTGACTGCCTCGGGTACTCTCACATCTAAGTCGGTTGGGTTGAACCTAACGACCCAGTTTGACGTACAGGACCGAGAGAGAACTTTTGGAATTGGGCGTAATGTCTACTTTGCTAGTCCGAGGTCCAGCTTCACGTCCATCaaca                                                                                                                                                                                                  >  1:510679/1‑301 (MQ=255)`  
                                                                                                                 `aCTTCAACTTCTACCCTGCGTCCATTGCGAACCTTAGTGATGACGACCCTATAGACGTAGCTGTGAGTACCAACCGAATAGCAATCCTTAAGTACGCCGTTCCGTTCTCAGAAGAGTTACTCATCTGGTCCGATGAAGCACAATTCGTCCTGACTGCCTCGGGTACTCTCACATCTAAGTCGGTTGAGTTGAACCTAACGACCCAGTTTGACGTACAGGACCGAGCGAGACCTTTTGGGATTGGGCGTAATGTCTACTTTGCTAGTCCGAGGTCCAGCTTCACGTCCATCCACAGGtacta                                                                                                                                                                                           <  1:608848/301‑1 (MQ=255)`  
                                                                                                                  `cTTCAACTTCTACCCTGCGTCCATTGCGAACCTTAGTGATGACGACCCTATAGACGTAGCTGTGAGTACCAACCGAATAGCAATCCTTAAGTACGCCGTTCCGTTCTCAGAAGAGTTACTCATCTGGTCCGATGAAGCACAATTCGTCCTGACTGCCTCGGGTACTCTCACATCTAAGTCGGTTTGGTTGAACCTAACGACCCAGTTTGACGTACAGGACCGAGCGAGACCTTTTGGGATTGGGCGTAATGTCTACTTTGCTAGTCCGAGGTCCATCTTCACGTCCATCCACAGGtactac                                                                                                                                                                                          >  1:21229/1‑301 (MQ=255)`  
                                                                                                                    `cagaCTTCTACCCGGCGTCCATTGCGAACCTTAGTGATGACGACCCTATAGAAGTAGCTGTGAGTACCAACCGAATAGCAATCCGTAAGTACACCTGTCCGTTCTCAGAAGAGTTACTCATCTGGTCCGATGAAGCACAATTCGTCCTGACTGCCTCGGGTACTCTCACATCTAAGTCGGTTGAGTTGAACCTAACGACCCAGTTTGACGTACAGGACCGAGCGAGACCTTTTGGGATTGGGCGTAATGTCTACTTTGCTAGTCCGAGGTCCAGCTTCACGTCCATCCACAGGTACTACGc                                                                                                                                                                                        <  1:301861/298‑1 (MQ=255)`  
                                                                                                                        `cTTCTACCCTGCGTCCATTGCGAACCTTAGTGATGACGACCCTATAGACGTAGCTGTGAGTACCAACCGAATAGCAATCCTTAAGTACGCCGTTCCGTTCTCAGAAGAGTTACTCATCTGGTCCGATGAAGCACAATTCGTCCTGACTGCCTCGGGTACTCTCACATCTAAGTCGGTTGGGTTGAACCTAACGACCCAGTTTGACGCACAGGACCGAGCGAGACCTTTTGGGATTGGGCGTAATGTCTACTTTGCTAGTCCGGGGTCCAGCTTCACGTCCATCCACAGTTACTACGCtgtg                                                                                                                                                                                    >  1:434730/1‑301 (MQ=255)`  
                                                                                                                               `ccTGCGTCCATTGCGAACCTTAGTGATGACGACCCTATAGACGTAGCTGTGAGTACCAACCGAAGAGCAATCCTTAAGTACGCCGTTCCGTTCTCAGAAGAGTTACTCATCTGGTCCGATGAAGCACAATTCGTCCTGACTGCCTCGGGTACTCTCACATCTAAGTCGGTTGGGTTGAACCTAACGACCCAGTTTGACGTACAGGACCGAGCGAGACCTTTTGGGATTGGGCGTAATGTCTACTTTGCTAGTCCGAGGTCCAGCTTCACGTCCATCCACAGGTACTACGCTGTGCAGGATg                                                                                                                                                                             <  1:602998/301‑1 (MQ=255)`  
                                                                                                                                      `taagagacagccTTAGTGATGACGACCCTATAGACGTAGCTGTGAGTACCAACCGAATATCAATCCTTAAGTACGCCGTTCCGTTCTCAGAAGAGTTACTCATCTGGTCCGATGAAGCACAATTCGTCCTGACTGCCTCGGGTACTCTCACATCTAAGTCGGTTGGGTTGAACCTAACGACCCAGTTTGACGTACAGGACCGAGCGAGACCTTTTGGGATTGGGCGTAATGTCTACTTTGCTAGTCCGAGGTCCAGCTTCACGTCCATCCACAGGTACTACGCTGTGCAGGATGTCAGTTc                                                                                                                                                                      <  1:542634/291‑1 (MQ=255)`  
                                                                                                                                         `ttGCGAACCTTAGTGATGACGACCCTATAGACGTAGCTGTGAGTACCAACCGAATAGCAATCCTTAAGTACGCCGTTCCGTTCTCAGAAGAGTTACTCATCTGGTCCGATGAAGCACAATTCGTCCTGACTGCCTCGGGTACTCTCACATCTAAGTCGGTTGGGTTGAACCTAACGACCCAGTTTGACGTACAGGACCGAGCACGACCTTTTGGGATTGGGCGTAATGTCTACTTTGCTAGTCCGAGGTCCAGCTTCACGTCCATCCACAGGTACTACGCTGTGCAGGATGTCAGTTCCGt                                                                                                                                                                   >  1:80866/1‑301 (MQ=255)`  
                                                                                                                                               `gtataagagacagcgacCCTAGAGACGTAGCTGTGAGTACCAACCGAATAGCAATCCTTAAGTACGCCGTTCCGTTCTCAGAAGAGTTACTCATCTGGTCCGATGAAGCACAATTCGTCCTGACTGCCTCGGGTACTCTCACATCTAAGTCGGTTGAGTTGAACCTAACGACCCAGTTTGACGTACAGGACCGAGCGAGACCTTTTGGGATTGGGCGTAATGTCTACTTTGCTAGTCCGAGGTCCAGCTTCACGTCCATCCACAGGTACTACGCTGTGCAGGATGTCAGTTCCGTTAAGaa                                                                                                                                                             <  1:231808/288‑1 (MQ=255)`  
                                                                                                                                                `ccTTAGTGATGACGACCCTATAGACGTAGCTGTGAGTACCAACCGAATAGCAATCCTTAAGTACGCCGTTCCGTTCTCAGAAGAGTTACTCATCTGGTCCGATGAATCACAATTCGTCCTGACTGCCTCGGGTACTCTCACATCTAAGTCGGTTGGGTTGAACCTAACGACCCAGTTTGACGTACAGGACCGAGCGAGACCTTTTGGGATTGGGCGTAATGTCTACTTTGCTAGTCCGAGGTCCAGCTTCACGTCCATCCACAGGTACTACGCTGTGCAGGATGTCAGTTCCGTTAAGAAt                                                                                                                                                            >  1:499075/1‑301 (MQ=255)`  
                                                                                                                                                `ccTTAGTGATGACGACCCTATAGACGTAGCTGTGAGTACCAACCGAATAGCAATCCTTAAGTACGCCGTTCCGTTCTCAGAAGAGTTACTCATCTGGTCCGATGAAGCACAATTCGTCCTGACTGCCTCGGGTACTCTCACATCTAAGTCGGTTGGGTTGAACCTAACGACCCAGTTTGACGTACAGGACCGAGCGAGACCTTTTGGGATTGGGCGTAATGTCTACTTTGCTAGTCCGAGGTCCAGCTTCACGTCCATCCACAGGTACTACGCTGTGCAGGATGTcctgtctcttatacac                                                                                                                                                            >  1:454808/1‑286 (MQ=255)`  
                                                                                                                                                       `gatgaCGACCCTATAGACGTAGCTGTGAGTACCAACCGAATAGCAATCCTTAAGTACGCCGTTCCGTTCTCAGAAGAGTTACTCATCTGGTCCGATGAAGCACAATTCGTCCTGACTGCCTCGGGTACTCTCACATCTAAGTCGGTTGAGTTGAACCTTACGACCCAGTTTGACGTACAGGACCGAGCGAGACCTTTTGGGATTGGGCGTAATGTCTACTTTGCTAGTCCGAGGTCCAGCTTACCGTCCATCCACAGGTACGACGCTGTGCAGGATGTCAGTTCCGTTAAGAATGCTGatg                                                                                                                                                     >  1:405006/1‑299 (MQ=255)`  
                                                                                                                                                         `tgaCGACCCTATAGACGTAGCTGTGAGTACCAACCGAATAGCAATCCTTAAGTACGCCGTTCCGTTCTCAGAAGAGTTACTCATCTGGTCCGATGAAGCACAATTCGTCCTGACTGCCTCGTGTACTCTCACATCTAAGTCGGTTGGGTTGAACCTAACGACCCAGTTTGACGTACAGGACCGAGCGAGACCTTTTGGGATTGGGCGTAATGTCTACTTTGCTAGTCCGAGGTCCAGCTTCACGTCCATCCACAGGTACTACGCTGTGCAGGATGTCAGTTCCGTTAAGAATGCTGAGGAc                                                                                                                                                   >  1:126424/1‑301 (MQ=255)`  
                                                                                                                                                         `aaacgacCCTATAGACGTAGCTGTGAGTACCAACCGAATAGCAATCCTTAAGTACGCCGTTCCGTTCTCAGAAGAGTTACTCATCTTGTCCGATGAAGCACAATTCGTCCTGACTGCCTCGGGTACTCTCACACCTAAGTCGGTTGAGTTGAACCTAACGACACAGTTTGACGTACAGGACCGAGCGAGACCTTTTGGGATTGGGCGTAATGTCTACTTGGCTAGTCCGAGGTCCAGCTTAACGGCCATCCACAGGTACTACGCTGTGCAGGATGTCAGTTCCGTTAAGAATGCTGAGGAc                                                                                                                                                   <  1:558545/299‑1 (MQ=255)`  
                                                                                                                                                              `tgtataagagataggCTGTGAGTACCAACCGAATAGCAATCCTTAAGTACGCCGTTCCGTTCTCAGAAGAGTTACTCATCTGGTCCGATGAAGCACAATTCGTCCTGACTGCCTCGGGTACTCTCACATCTGAGTCGGTTGGGTTGAACCTAACGACCCAGTTTGACGTACAGGACCGAGCGAGACCTTTTGGGATTGGGCGTAATGTCTACTTTGCTAGTCCGAGGTCCAGCTTCACGTCCATCCACAGGTACTACGCTGTGCAGGATGTCAGTTCCGTTAAGAATGCTGAGGACATTAc                                                                                                                                              <  1:25406/287‑1 (MQ=255)`  
                                                                                                                                                              `ccccAATAGACGTAGCTGTGAGTACCAACCGAATAGCAATCCTTAAGTACGCCGTTCCGTTCTCAGAAGAGTTACTCATCTGGTCCGATGAAGCACAATTCGTCCTGACTGCCTCGGGTACTCTCACATCTAAGTCGGTTGGGTTGAACCTAACGACCCAGTTTGACGTACAGGACCGAGCGAGACCTTTTGGGATTGGGCGTAATGTCTACGTTGCTAGTCCGAGGTCCAGCTTCACGTCCATCCACAGGTACTACGCTGTGCAGGATGTCAGTTCCGTTAATAATGCTGAGGACATTAc                                                                                                                                              <  1:647407/300‑1 (MQ=255)`  
                                                                                                                                                              `agacTATAGATGTAGCTGTGAGTACCAACCGAATAGCAATCCTTAAGTACGCCGTTCCGTTCTCAGAAGAGTTACTCATCTGGTCCGATGAAGCACAATTCGTCCTGACTGCCTCGGGTACTCTCACATCTAAGTCGGTTGGGTTGAACCTAACGACCCAGTTTGACGTACAGGACCGAGCGAGACCTTTTGGGATTGGGCGTAATGTCTAATTTGCTAGTCCGAGGTCCAGCTTCACGTCCATCCACAGGTACTACGCTGTGCAGGATGTCAGTTCCGTTAAGAATGCTGAGGACATTAc                                                                                                                                              <  1:146008/298‑1 (MQ=255)`  
                                                                                                                                                              `acCCTATAGACGTAGCTGTGAGTACCAACCGAATAGCAATCCTTAAGTACGCCGTTCCGTTCTCAGAAGAGTTACTCATCTGGTCCGATGAAGCACAATTCGTCCTGACTGCCTCGGGTACTCTCACATCTAAGTCGGTTGGGTTGAACCTAACGACCCAGTTTGACGTACAGGACCGAGCGAGACCTTTTGGGATTGGGCGTAATGTCTACTTTGCTAGTCCGAGGTCCAGCTTCACGTCCATACACAGGTACTACGCTGTGCAGGATGTCAGTTCCGTTAAGAATGCTGAGGACATTAc                                                                                                                                              >  1:222094/1‑301 (MQ=255)`  
                                                                                                                                                              `aagagacaGACGTAGCTGTGAGTACCAACCGAATAGCAATCCTTAAGTACGCCGTTCCGTTCTCAGAAGAGTTACTCATCTGGTCCGATGAAGCACAATTCGTCCTGACTGCCTCGGGTACTCTCACATCTAAGTCGGTTGAGTTGAACCTAACGACCCAGTTTGACGTACAGGACCGAGCGAGACCTTTTGGGATTGGGCGTAATGTCTACTTTGCTAGTCCGAGGTCCAGCTTCACGTCCATCCACAGGTACTACGCTGTGCAGGATGTCAGTTCCGTTAAGAATGCTGAGGACATTAc                                                                                                                                              <  1:529274/294‑1 (MQ=255)`  
                                                                                                                                                                `aacagaGACGTAGCGGTGAGTACCAACCGAATAGCAATCCTTAAGTACGCCGTTCCGTTCTCAGAGGATTTACTCATCTGGTCCGATGAAGCACAATTCGTCCTGACTGCCTCGGGTACTCTCACATCTAAGTCGGTTGAGTTGAACCTAACGACCCAGTGTGACGTACAGGACCGAGCGAGACCTTTTGGGATTGGGCGTAATGTCTACTTTGCTAGTCCGAGGTCCAGCTTCACGTCCATCCACAGGTACTACGCTGTGCAGGATGTCAGTTCCGTTAAGAATGCTGAGGGCATTACAt                                                                                                                                            <  1:481907/296‑1 (MQ=255)`  
                                                                                                                                                                   `ataGACGTAGCTGTGAGTACCAACCGAATAGCAATCCTTAAGTACGCCGTTCCGTTCTCAGAAGAGTTACTCATCTGGTCCGATGAAGCACCATTCGTCCTGACTGCCTCGGGTACTCTCACATCTAAGTCGGTTGGGTTGAACCTAACGACCCAGTTTGACGTACAGGACCGAGCGAGACCTTTTGGGATTGGGCGTAATGTCTACTTTGCTAGTCCGAGGTCCAGCTTCACGTCCATCCACAGGTACTACGCTGTGCAGGATGTCAGTTCCGTTAAGAATGCTGAGGACATTACATcac                                                                                                                                         <  1:476209/301‑1 (MQ=255)`  
                                                                                                                                                                     `aGACGTAGCTGTGAGTACCAACCGAATAGCAATCCTTAAGTACGCCGTTCCGTTCTCAGAAGAGTTACTCATCTGGTCCGATGAAGCACAATTCGTCCTGACTGCCTCGGGTACTCTCACATCTAAGTCGGTTGGGTTGAACCTAACGACCCAGTTTGACGTACAGGACCGAGCGAGACCTTTTGGGATTGGGCGTAATGTCTACTTTGCTAGTCCGAGGTCCAGCTTCACGTCCATCCACAGGTACTACGCTGTGCAGGATGTCAGTTCCGTTAAGAATGCTGAGGACATTACATCacac                                                                                                                                       >  1:150343/1‑301 (MQ=255)`  
                                                                                                                                                                     `aGACGTAGCTGTGAGTACCAACCGAATAGCAATCCTTAAGTACGCCGTTCCGTTCTCAGAAGAGTTACTCATCTGGTCCGATGAAGCACAATTCGTCCTGACTGCCTCGGGTACTCTCACATCTAAGTCGGTTGGGTTGAACCTAACGACCCAGTTTGACGTACAGGACCGAGCGAGACCTTGTGGGATTGGGCGTAATGTCTACTTTGCTAGTCCGAGGTCCAGCTTCACGTCCATCCACAGGTACTACGCTGTGCAGGATGTCAGTTCAGTTAAGAATGCTGAGGACATTACATCacac                                                                                                                                       <  1:516888/301‑1 (MQ=255)`  
                                                                                                                                                                            `gCTGTGAGTACCAACCGAATAGCAATCCTTAAGTACGCCGTTCCGTTCTCAGAAGAGTTACTCATCTGGTCCGATGAAGCACAATTCGTCCTGACTGCCTCGGGTACTCTCACAACTAAGTCGGTTGCGTTGAACCTAACGACCCAGTTTGACGTACAGGACCGAGCGAGACCTTTTGGGATTGGGCGTAATGGCTACTTTGCTAGTCCGAGGTCCAGCTTCACGTCCATCCACAGGTACTACGCTGTGCAGGATGTCAGTTCCGTTAAGAATGCTGAGGAcctgtctcttatacacatct                                                                                                                                >  1:442256/1‑282 (MQ=255)`  
                                                                                                                                                                              `tgtgAGTACCAACCGAATAGCAATCCTTAAGTACGCCGTTCCGTTCTCAGAAGAGTTACTCATCTGGTCCGATGAAGCACAATTCGTCCTGACTGCCTCGGGTACTCTCACATCTAAGTTGGTTGGGTTGAACCTAAAGACCCAGTTTGACGTACAGGACCGAGCGAGACCTTTTGGGCTTGGGCGTAATGTCTACTTTGCTAGTCCGAGGTCCAGCTTCACGTCCATCCACAGGTACTACGCTTTGCAGGATGTAAGTTCCGTCAAGAATGCTTAGGACATTACATCACACGTTCCTAAc                                                                                                                              >  1:667436/1‑301 (MQ=255)`  
                                                                                                                                                                               `gtgAGTACCAACCGAATAGCAATCCTTAAGTACGCCGTTCCGTTCTCAGAAGAGTTACTCATCTGGTCCGATGAAGCACAATTCGTCCTGCCTGCCTCGGGTACTCTCACATCTAAGTCGGTTGGGTTGAACCTAACGACCCAGTTTGACGTACAGGACCGAGCGAGACCTTTTGGGATTGGGCGTAATGTCTACTTTGCTAGTCCGAGGTCCAGCTTCACGTCCATCCACAGGTACTACGCTGTGCAGGATGTCAGTTCCGTTAAGAATGCTGAGGACATTACATCACACGTTCCTAACt                                                                                                                             <  1:179890/301‑1 (MQ=255)`  
                                                                                                                                                                               `gtgAGTACCAACAGAATAGCATTCCGTAAGTACGCCGTTCCGTTCTCAGAAGAGTTGCTCATCTGTTCCGATGATGCTCAATTCGTCCTGACTGCCTCGGGTACTCTCACATCTAAGTCGTTTGGCTTGAACCTAACGACCCAGTTTGTCGTACAGGACCGAGCGAGACCTTTTGGGATTGGGCGTAATGTCTACTTTGCTAGTCCGAGGTCCAGCTTCACGTCCATCCACAGGTATTACTCTGTGCAGGATGTCAGTTCCGTTAAGAATGCTGAGGACATTACATCACACGTTCCTAACt                                                                                                                             <  1:655158/301‑1 (MQ=255)`  
                                                                                                                                                                                `ttaGTACCAACCGAATAGCAATCCTTAAGTACGCCGTTCCGTTCTCAGAAGAGTTACTCATCTGGTCCGATGAAGCACAATTCGTCCTGACTGCCTCGGGTACTCTCACATCTAAGTCGGTTGGGTTGAACCTAACGACCCCGTTTGACGTACAGGACCGAGCGAGACCTTTTGGGATTGGGCGTAATGTCTACTTTGCTAGTCCGAGGTCCAGCTTCACGTCCATCCACAGGTACTACGCTGTGCAGGATGTCAGTTCCGTTAAGAATGCTGAGGACATTACATCACACGTTCCTAACTa                                                                                                                            <  1:308301/299‑1 (MQ=255)`  
                                                                                                                                                                                 `gAGTACCAACCGAATAGCAATCCTTAAGTACGCCGTTCCGTTCTCAGAAGAGTTACTCATCTGGTCCGATGAAGCACAATTCGTCCTGACTGCCTCGGGTACTCTCACATCTAAGTCGGTTGAGTTGAACCTAACGACCCAGTTTGACGTACAGGACCGAGCGAGACCTTTTGGGATTGGGCGTAATGTCTACTTTGCTAGTCCGAGGTCCAGCTTCACGTCCATCCACAGGTACTACGCTGTGCAGGATGTCAGTTCCGTTAAGAATGCTGAGGCCATTACATCACACGTTCCTAACTAc                                                                                                                           <  1:237487/301‑1 (MQ=255)`  
                                                                                                                                                                                 `aaGTAACAACCGAAGAGCAATCATTAAGTACGCCGTTCCGTTCTCAGAAGAGTTACTCATCTGGTCCGAAGAAGCACAATTCGTCCTGACTGCCTCGGGTAATCTCACATCTAAGTCGGTTGAGTTGAACCTAACGACCCAGTTTGACGTACAGGACCGAGCGAGACCTTTTGGGATTGGGCGTAATGTCTACTTTGCTAGTCCGAGGTCCAGCTTCACGGCCATCCACAGGTACTACGCTGTGCAGGATGTCAGTTCCGTTAAGAATGCTGAGGACATTACATCACACGTTCCTAACTAc                                                                                                                           <  1:385716/300‑1 (MQ=255)`  
                                                                                                                                                                                  `aGTACCAACCGAATAGCAATCCTTAAGTACGCCGTTCCGTTCTCAGAAGAGTTACTCATCTGGTCCGATGAAGCACAATTCGTCCTGACTGCCTCGGGTACTCTCACATCTAAGTCGGTTGGGTTGAACCTAACGACCCAGTTTGACGTCCAGGACCGAGCGAGACCTTTTGGGATTGGGCGTAATGTCTACTTTGCTAGTCCGAGCTCCAGCTTCACGTCCATCCACAGTTACTACGCTGTGCAGGCTGTCAGTTCCGTTCAGAATGCTGAGGACATTACATCCCACGTTCCTAACTCCa                                                                                                                          >  1:379052/1‑301 (MQ=255)`  
                                                                                                                                                                                   `gTACCAACCGAATAGCAATCCTTAAGTACGCCGTTCCGTTCTCAGAAGAGTTACTCATCTGGTCCGATGAAGCACAATTCGTCCTGACTGCCTCGGGTACTCTCACATCTAAGTCGGTTGGGTTGAACCTAACGACCCAGTTTGACGTACAGGACCGAGCGAGACCTTTTGGGATTGGGCGTAATGTCTACTTTGCTAGTCCGAGGTCCAGCTTCACGTCCATCCACAGGTACTACGATGTTCAGGATGTCAGTTCCGTTAAGCATGCTGAGGACATTACATcctgtctcttatacacatc                                                                                                                         >  1:625863/1‑283 (MQ=255)`  
                                                                                                                                                                                      `ggtataagagacagaTCCTTAAGTACGCCGTTCCTTTCT‑AGAAGAGTTACTCATCTGGTCCGATGAAGCACAATTCGTCCTGACTGCCTCGGGTACTCTCACATCTAAGTCGGTTGGGTTGAACCTAACGACCCAGTTTGACGTACAGGACCGAGCGAGACCTTTTGGGATTGGGCTTAATGTCTACTTTGCTAGTCCGAGGTCCAGCTTCACGTCCATCCACAGGTACTACGCTGTGCAGGATGTCAGTTCCGTTAAGAATGCTGAGGACATTACATCACACGTTCCTAACTACATCCCt                                                                                                                     <  1:329663/287‑1 (MQ=255)`  
                                                                                                                                                                                       `cAACTGAATAGCAATCCTTAAGTACGCCGTTCCGTTCTCAGAAGAGTTACTCATCTGGTCCGATGAAGCACAATTCGTCCTGACTGCCTCGGGTACTCTCACATCTAAGTCGGTTGGGTTGAACCTAACGACCCAGTTTGACGTACAGGACCGAGCGAGACCTTTTGGGATTGGGCGTAATGTCTACTTTGCTAGTCCGAGGTCCAGCTTCACGTCCATCCACAGGTACTACGCTGTGCAGGATGTCAGTTCCGTTAAGAATGCTGAGGACATTACATCCCCCGTTCCTAACTACATCCCt                                                                                                                     >  1:31571/1‑301 (MQ=255)`  
                                                                                                                                                                                       `cAACCGAATAGCAATCCTTAAGTACGCCGTTCCGTTCTCAGAAGAGTTACTCATCTGGTCCGATGAAGCACAATTCGTCCTGACTGCCTCGGGTACTCTCACATCTAAGTCGGTTGGATTGAACCTAACGACCCAGTTTGACGTACAGGACCGAGCGAGACCTCTTGGGATTGGGCGTAATGTATACTTTGCTAGTCCGAGGTCCAGCTTCACGTCCATCCACAGGTACTACGCTGTGCAGGATGTCAGTTCCGTTAAGAAGACTGAGGAACTTACATACAAGGTTCTTAAATACATCCCt                                                                                                                     >  1:585808/1‑301 (MQ=255)`  
                                                                                                                                                                                            `gAATAGCAATCCTTAAGTGCGCCGTTCCGTTCTCAGAAGAGTTACTCATCTGGTCCGATGAAGCACAATTCGTCCTGACTGCCTCGGGTACTCTCACAGCTAAGTCGGTTGCGTTGAACCTAACGACCCAGTTTGACGTACAGGACCGAGCGAGACCTTTTGGGATTGGGCGTAATGTCTACTTTGCTAGTCCGAGGTCCAGCTTCACGTCCATCCACAGGTACTACGCTGTGCAGGATGTCAGTTCCGTTAAGAATGCTGAGGACATTACATCACACGTTCCTAACTACATCCCTAATgg                                                                                                                <  1:272548/301‑1 (MQ=255)`  
                                                                                                                                                                                                `aGCAATCCTTAAGTACGCCGTTCCGTTCTCAGAAGAGTTACTCATCTGGTCCGATGACGCACAATTCGTCCTGACTGCCTCGGGTACTCTCACATCTAAGTCGGTTGGGTTGAACCTAACGACCCAGTTTTACGTCCAGGACCGAGCGGGGCCTTTTGGGATTGGGCGTAATGTCTACTTTGCTAGTCCGAGGTCCCGCTTCTCGTCCATCCACGGGTACTACGCTGTGCAGGATGTCAGTTCCGTTACGAATGCTGAGGACATTACATCACACGTTCCTAACTACCTCCCTAATGgtgtt                                                                                                            >  1:563420/1‑300 (MQ=255)`  
                                                                                                                                                                                                     `tCCTTAAGTACGCCGTTCCGTTCTCAGAAGAGTTACTCATCTGGTCCGATGAAGCACAATTCGTCCTGACTGCCTCGGGTACTCTCACATCTAAGTCGGTTGGGTTGAACCTAACGACCCAGTTTGACGTACAGGACCGCGCGAGACCTTTTGGGATTGGGCGTAATGTCTACTTTGCTAGTCCGAGGTCCAGCTTCACGTCCATCCACAGGTACTACGCTGTGCAGGATGTCAGTTCCGTTAAGAATGCTGAGGACATTACATCACACGTTCCTAACTACATCCCTAATGGTGTTTTcct                                                                                                       >  1:46019/1‑299 (MQ=255)`  
                                                                                                                                                                                                     `tCCTTAAGTACGCCGTTCCGTTCTCAGAAGAGTTACTCATCTGGTCCGATGAAGCACAATTCGTCCTGACTGCCTCGGGTACTCTCACATCTAAGTCGGTTGGGTTGAACCTAACGACCCAGTTTGACGTACAGGACCGAGCGAGACCTTTTGGGATTGGGCGTAATGTCTACTTTGCTAGTCCGAGGTCCAGCTTCACGTCCATCCACAGGTACTACGCTGTGCAGGATGTCAGTTCCGTTAAGAATGCTGAGGACATTACATCACACGTTCCTAACTACATCCCTAATGGTGTGTTcct                                                                                                       >  1:580746/1‑299 (MQ=255)`  
                                                                                                                                                                                                      `ccTTAAGTACGCCGTTCCGTTCTCAGAAGAGTTACTCATCTGGTCCGATGAAGCACAATTCGTCCTGACTGCCTCGGGTACTCTCACATCTAAGTCGGTTGGGTTGAACCTAACGACCCAGTTTGACGTACAGGACCGAGCGAGACCTTTTGGGATTGGGCGTAATGTCTACTTTGCTAGTCCGAGGTCCAGCTTCACGTCCATCCACAGGTACTACGCTGTGCAGGATGTCAGTTCCGTTAAGAATGCTGAGGACATTACATCACACGTTCCTAACTACATCCCTAATGGTGTGTTCAGt                                                                                                      >  1:137092/1‑301 (MQ=255)`  
                                                                                                                                                                                                        `ttAAGTACGCCGTTCCGTTCTCAGAAGAGTTACTCATCTGGTCCGATGAAGCACAATGCGTCCTGACTGCCTCGGGTACTCTCACATCTAAGTCGGTTGGGTTGAACCTAACGACCCAGTGTGACGTACAGGACCGAGCGAGACCTTTTGGGATTGGGCGTAACGTCTACTTTGCTAGTCCGAGGTCCAGCTTCACGTCCATCCACAGGTACTACGCTGTGCAGGATGTCAGTTCCGTTAAGAATGCTGAGGACATTACATCACACGTTCCTAACTACATCCCTAATGGTGTGTTCAGTAt                                                                                                    <  1:288729/301‑1 (MQ=255)`  
                                                                                                                                                                                                        `ttAAGTACGCCGTTCCGTTCGCAGAAGAGTTACTCATCTGGTCCGATGAAGCACAATTCGTTCTGACTGCCTCGGGTACTCTCACATCTAAGTCGGTTGGGTTGAACCTAACGACCCAGTTTGACGTACAGGACCGAGCGAGACCTTTTGGGATTGGGCGTAATGTCTACTTTGCTAGTCCGAGGTCCAGCTTCACGTCCGTCCACAGGTACTACGCTGTGCAGGATGTCAGTTCCGTTAAGAATGCTGAGGACATTACATCACACGTTCCTAACTACATCCCTAATGGTGTGTTCAGTAt                                                                                                    <  1:75697/301‑1 (MQ=255)`  
                                                                                                                                                                                                        `cagtgTACGCCGTTCCGTTCTCAGAAGAGTTACTCATCTGGTCCGATGAAGCACAATTCGTCCTGACTGCCTCGGGTACTCTCACATCTAAGTCGGTTGGGTTGAACCTAACGACCCAGTTTGACGTACAGGACCGCGCGAGACCTTTTGGGATTGGGCGTAATGTCTACTTTCCTAGTCCGAGGTCCAGCTTCACGTCCATCCACAGGTACTACGCTGTGCAGGATGTCAGTTCCGTTAAGAATGCTGAGGACATTACATCACACGTTCCTAACTACATCCCTAATGGTGTGTTCAGTAt                                                                                                    <  1:87200/297‑1 (MQ=255)`  
                                                                                                                                                                                                         `tAAGTACGCCGTTCCGTTCTCAGAAGAGTTACTCATCTGGTCCGATGAAGCACAATTCGTCCTGACTGCCTCGGGTACTCTCACATCTAAGTCGGTTGGGTTGAACCTAACGACCCAGTTTGACGTACAGGACCGAGCGTGACCTTTTGGGATTGGGCGTAATGTCTACTTTGCTAGTCCGAGGTCCAGCTTCACTTCCATCCACAGGTACTACGATGTGCATTATGTCAGTTCCGTTAAGAATGCTGAGGACATTACATTACACGTTCCTAACTACATCCCTAATGGTTTGTTTAGTAtt                                                                                                   >  1:259043/1‑301 (MQ=255)`  
                                                                                                                                                                                                         `tAAGTACGCCGTTCCGTTCTCAGAAGAGTTACTCATCTGGTCCGATGAAGCACAATTCGTCCTGACTGCCTCGGGTACTCTCACATCTAAGTCGGTTGGGTTGAACCTAACGACCCAGTTTGACGTACAGGACCGAGCGAGACCTTTTGGGATTGGGCGTAATGTCTACTTTGCTAGTCCGAGGTCCAGCTTCACGTCCATCCACAGGTACTACGCTGTGCAGGATGTCAGTTCCGTTAAGAATGCTGAGGTCATTACATCACACGTTCCTAACTACATCCCTAATGGTGTGTTCCGtctg                                                                                                   >  1:464831/1‑298 (MQ=255)`  
                                                                                                                                                                                                              `cggagatgtgtataagagacaggTTACTCATCTGGTCCGATGAAGCACAATTCGTCCTGACTGCCGCGGGTACTCTCACATCTAAGTCGGTTGAGTTGAACCTAACGACCCAGTTTGACGTACAGGACCGAGCGAGACCTTTTGGGATTGGGCGTAATGTCTACTTTGCTAGTCCGAGGTCCAGCTTCACGTCCATCCACAGGTACTACGCTGTGCAGGATGTCAGTTCCGTTAAGAATGCTGAGGACATTACATCACACGTTCCTAACTACATCCCTAATGGTGTGTTCAGTATTTGCgg                                                                                              <  1:523043/279‑1 (MQ=255)`  
                                                                                                                                                                                                              `aCGCCGTTCCGTTCTCAGAATAGTTACTCATCTGGTCCGATGAAGCACAATTCGTCCTGACTGCCTCGGGTACTCTCACATCTAAGTCGGTTGGGTTTAACCTAACGACCCAGTTTGACGTACAGGACCGAGCGAGACCTTTTGGGTTTGGGCGTAATGTCTACTTTGCTAGTCCGCGGTCCCGCTTCACGTCCCTCCACAGGTACTACGCTGTGCCGGATGTCAGTTCCGTTAAGAATGCTGAGGCCATTACATCACACGTTCCTAACTACTTCCCTAAGGGTGTGTTCAGTATTTGCgg                                                                                              >  1:618780/1‑301 (MQ=255)`  
                                                                                                                                                                                                              `aCGCCGTTCCGTTCTCAGAAGAGTTACTCATCTGGTCCGATGAAGCACAATTCGTCCTGACTGCCTCGGGTACTCTCACATCTAAGTCGGTTGGGTTGAACCTAACGACCCAGTTTGACGTACAGGACCGAGCGAGACCTTTTGGGATTGGGCGTACTGTCTACTTTGCTAGTCCGAGGTCCAGCTTCACGTCCATCCACAGGTACTACGCTGTGCAGGATGTCAGTTCCGTTAAGAATGCTGAGGACATTACATCACACGTTCCTAACTCCATCCCTAATGGTGTGTTCATTATTTGCgg                                                                                              >  1:448590/1‑301 (MQ=255)`  
                                                                                                                                                                                                                   `gttccgttcTCAGAAGAGTTACTCATCTGGTCCGATGAAACACAATTCGTCCTGACTGCCTCGGGTACTCTCACATCTAAGTCGGTTGGGTTGAACCTAACGACCCAGTTTGACGTACAGGACCGAGCGAGACCTTTTGGGATTGGGCGTAATGTCTACTTTGCTAGTCCGAGGTCCAGCTTCACGTCCATCCACAGGTACTACGCTGTGCAGGATGTCAGTTCCGTTACGAATGCTGAGGTCATTACATCACACGTTCCTAACTACATCCCTAATGGTGTGTTCAGTATTTGCGGAAGTg                                                                                         >  1:613569/1‑301 (MQ=255)`  
                                                                                                                                                                                                                    `ttccgttcTCAGAAGAGTTACGCATCTGGTCCGATGAAGCACAATTCGTCCTGACTGCCTCGGGTACTCTCACATCTAAGTCGGTTGGGTTGAACCTAACGACCCAGTTTGACGTACAGGACCGAGCGAGACCTTTTGGGATTGGGCGTAATGTCTACTTTGCTAGTCCGAGGTCCAGCTTCACGTCCATCCACAGGTACTACGCTGTGCAGGATGTCAGTTCCGTTAAGAATGCTGAGGACATTACATCACACGTTCCTAACTACATCCCTAATGGTGTGTTCAGTATTTGCGGAAGTgg                                                                                        <  1:226670/301‑1 (MQ=255)`  
                                                                                                                                                                                                                                  `gagTTACTCATCTGGTCCGATGAAGCACAATTCGTCCTGACTGCCTCGGGTACTCTCACATCTAAGTCGGTTGGGTTGAACCTAACGACCCAGTTTGACGTACAGGACCGAGCGAGACCTTTTGGGATTGGGCGTAATGTCTACTTTGCTAGTCCGAGGTCCAGCTTCACGTCCATCCACAGGTACTACGCTGTGCAGGATGTCAGTTCCGTTAAGAATGCTGAGGACATTACATCACACGTTCCTAACTACATCCCTAATGGTGTGTTCAGTATTTGCGGAAGTGGTACGGAAAACTTCt                                                                          >  1:506074/1‑301 (MQ=255)`  
                                                                                                                                                                                                                                      `tagtcagcagggccGATGAAGCGCAATTCGACCTGACTGCATCGGGACATCTAAAATCTAAGGCGGTTGGGTTGAACCGAAAGAGCCAGTTTGACGTACAGGACCGAGCGAGGCCTTTTGGGATTGAGCGTAATGTCTACTTTGCTAGTCCGAGGTCCAGCTTCGCGTCCATCCACAGGTACTACGCTGTGCAAGATGTCAGTTCCGTTAAAAATGCTGAGGACATTACATCACACGTTCCTAACTACATACCTAATGGTGTGTTCAGTATTTGCGGAAGTGGTACGGAAAACTTCTGTTc                                                                      <  1:425883/289‑1 (MQ=255)`  
                                                                                                                                                                                                                                          `cATCTGGTCCGATGAAGCACAATTCGTCCTGACTGCCTCGGGTACTCTCACATCTAAGTCGGTTGGGTTGAACCTAACGACCCAGTTTGACGTACAGGACCGAGCGAGACCTTTTGGGATTGGGCGTAATGTCTACTTTGCTAGTCCGAGGTCCAGCTTCACGTCCATCCACAGGTCCTACGCTGTGCAGGATGTCAGTTCCGTTAAGAATGCTGAGGACATTACATCACCCGTTCCTAACTACATCCCTAATGGTGTGTTCAGTATTTGCGGAAGTGGTACGGAAAACTTCTGTTCGGtt                                                                  >  1:173222/1‑300 (MQ=255)`  
                                                                                                                                                                                                                                          `cATCTGGTCCGATGAAGCACAATTCGTCCTGACTGCCTCGGGTACTCTCACATCTAAGTCGGTTGGGTTGAACCTAACGACCCAGTTTGACGTACAGGACCGAGCGAGACCTTTTGGGATTGGGCGTAATGTCTACTTTGCTAGTCCGAGGTCCAGCTTCACGTCCATCCACAGGTACTACGCTGTGCAGGATGTCAGTTCCGTTAAGAATGCTGAGGACATTACATCACACGTTCCTAACTACATCCCTAATGGTGTGTTCAGTA‑TTGCGGAAGTGGTACGGAAACCTTCTGTTCGGTAc                                                                 >  1:242912/1‑301 (MQ=255)`  
                                                                                                                                                                                                                                          `cATCTGGTCCGATGAAGCACAATTCGTCCTGACTGCCTCGGGTACTCTCACATCTAAGTCGGTTGCGTTGAACCTAACGACCCAGTTTGACGTACAGGACCGAGCGAGACCTTTTGGGATTGGGCGTAATGTCTACTTTGCTAGTCCGAGGTCCAGCTTCACGTCCATCCACAGGTACTACGCTGTGCAGGATGTCAGTTCCGTTAAGAATGCTGAGGACATTACATCACACGTTCCTAACTACATCCCTAATGGTGTGTTCAGTATTTGCGGAAGTGGTACGGAAAACTTCTGTTCGGTa                                                                  >  1:155619/1‑301 (MQ=255)`  
                                                                                                                                                                                                                                           `aTCTGGTCCGATGAAGCACAATTCGTCCTGACTGCCTCGGGTACTCTCACATCTAAGTCGGTTGGGTTGAACCTAACGACCCAGTTTGACGTACAGGACCGAGCGAGACCTTTTGGGATTGGGCGTAATGTCTACTTTGCTAGTCCGAGGTCCAGCTTCACGTCCATCCACAGGTACTACGCTGTGCAGGATGTCAGTTCCGTTAAGAATGCTGAGGCCATTACATCACACGTTCCTAACTACATCCCTAATGGTGTGTTCAGTATTTGCGGAAGTGGTACGGAAAACTTCTGTTCGGTAc                                                                 <  1:568160/301‑1 (MQ=255)`  
                                                                                                                                                                                                                                             `cTGGTACGATGAAGCACAATTCGTCCTGACTGCCTCGGGTA‑‑CTCACATCTAAGTCGGTTGAGTTGAACCTAACGACCCAGTTTGACGTACAGGACCGAGCGAGACCTTTTGGGATTGGGCGTAATGTCTACTTTGCTAGTCCGAGGTCCAGCTTCACGTCCATCCACAGGTACTACGCTGTGCAGGATGTCAGTTCCGTTAAGAATGCTGAGGACATTACATCACACGTTCCTAACTACATCCCTAATGGTGTGTTCAGTATTTGCGGAAGTGGTACGGAAAACTTCTGTTCGGTACTAtc                                                             <  1:166784/301‑1 (MQ=255)`  
                                                                                                                                                                                                                                                    `gATGAAGCACAATTCGTCCTGACTGCCTCGGGTACTCTCACATCTAAGTCGGTTGGGTTGAACCTAACGACCCAGTTTGACGTACAGGACCGAGCGAGACCTTTTGGGATTGGGCGTAATGTCTACTTTGCTAGTCCGAGGTCCAGCTTCACGTCCATCCACAGGTACTACGCTGTGCAGGATGTCAGTTCCGTTAAGAATGCTGAGGACATTACATCACACGTTCCTAACTACATCCCTAATGGTGTGTTCAGTATTTGCGGAAGTGGTACGGAAAACTTCTGTTCGTTACTATCTCACg                                                        >  1:107366/1‑301 (MQ=255)`  
                                                                                                                                                                                                                                                     `aTGAAGCACAATTCGTCCTGACTGCCTCGGGTACTCTCACATCTAAGTCGGTTGGGTTGAACCTAACGACCCAGTTTGACGTACAGGACCGAGCGAGACCTTTTGGGATTGGGCGTAATGTCTACTTTGCTAGTCCGAGGTCCAGCTTCACGTCCATCCACAGGTACTACGCTGTGCAGGATGTCAGTTCCGTTAAGAATGCTGAGGACATTACATCACACGTTCCTAACTACATCCCTAATGGTGTGTTCAGTATTTGCGGAAGTGGTACGGAAAACTTCTGTTCGGTACTATCTCACgg                                                       <  1:336412/301‑1 (MQ=255)`  
                                                                                                                                                                                                                                                       `gAAGCACAATTCGTCCTGACTGCCTCGGGTACTCTCACATCTAAGTCGGTTGGGTTGAACCTAACGACCCAGTTTGACGTACAGGACCGAGCGAGACCTTTTGGGATTGGGCGTAATGTCTACTTTGCTAGTCCGAGGTCCAGCTTCACGTCCATCCACAGGTACTACGCTGTGCAGGATGTCAGTTCCGTTAAGAATGCTGAGGACATTACATCACACGTTCCTAACTACATCCCTAATGGTGTGTTCAGTATTTGCGGAAGTGGTACGGAAAACTTCTGTTCGGTACTATCTCACgggg                                                     <  1:195434/301‑1 (MQ=255)`  
                                                                                                                                                                                                                                                         `aGCACAATTCGTCCTGACTGCCTCGTGTACTCTCACATCTAAGTCGGTTGGGTTGAACCTAACGACCCAGTTTGACGTACAGGACCGAGCGAGACCTTTTGGGATTGGGCGTAATGTCTACTTTGCTAGTCCGAGGTCCAGCTTCACGTCCATCCACAGGTACTACGCTGTGCAGGATGTCAGTTCCGTTAAGAATGCTGAGGACATTACATCACACGTTCCTAACTACATCCCTAATGGTGTGTTCAGTATTTGCGGAAGTGGTACGGAAAACTTCTGTTCGGTACTATCTCACGGGGAc                                                   >  1:633646/1‑301 (MQ=255)`  
                                                                                                                                                                                                                                                              `aaTTCGTCCTGACTGCCTCGGGTACTCTCACATCTAAGTCGGTTGGGTTGAACCTAACGACCCAGTTTGACGTACAGGACCGAGCGAGACCTTTTGGGATTGGGCGTAATGTCTACTTTGCTAGTCCGAGGTCCAGCTTCACGTCCATCCACAGGTACTACGCTGTGCAGGATGTCAGTTCCGTTAAGAATGCTGAGGACATTACATCACACGTTCCTAACTACATCCCCAATGGTGTGTTCAGTATTTGCGGAAGTGGTACGGAAAACTTCTGTTCGGTACTATCTCACGGGGACCCTAg                                              >  1:93448/1‑301 (MQ=255)`  
                                                                                                                                                                                                                                                                 `tCGTCCTGACTGCCTCGGGTACTCTCACATCTAAGTCGGTTGGGTTGAACCTAACGACCCAGTTTGACGTACAGGACCGAGCGAGACCTTTTGGGATTGGGCGTAATGTCTACTTTGCTAGTCCGAGGTCCAGCTTCACGTCCATCCACAGGTACTACGCTGTGCAGGATGTCAGTTCCGTTAAGAATGCTGAGGACATTACATCACACGTTCCTAACTACATCCCTAATGGTGTGTTCAGTATTTGCGGAAGTGGTACGGAAAACTTCTGTTCGGTACTATCTCACGGGGACCCTAGTaa                                           <  1:277706/301‑1 (MQ=255)`  
                                                                                                                                                                                                                                                                     `atataagagacagggTACTCTCACATCTAAGGCGGTTGGGTTGAACCTAACGACCCAGTTTGACGTACAGGACCGAGCGAGACCTTTTGGGATTGGGCGTAATGTCTACTTTGCTAGTCCGAGGTCCAGCTTCACGTCCATCCACAGGTACTACGCTGTGCAGGATGTCAGTTCCGTTAAGAATGCTGAGGACATTACATCACACGTTCCTAACTACATCCCTAATGGTGTGTTCAGTATTTGCGGAAGTGGTACGGAAAACTTCTGTTCGGTACTATCTCACGGGGACCCTAGTAAAATc                                       <  1:419165/289‑1 (MQ=255)`  
                                                                                                                                                                                                                                                                            `gCCTCGGGTACTCTCACATCTAAGTCGGTTGGGTTGAACCTAACGACCCAGTTTGACGTACAGGACCGAGCGAGACCTTTTGGGATTGGGCGTAATGTCTACTTTGCTAGTCCGAGGTCCAGCTTCACGTCCATCCACAGGTACTACGCTGTGCAGGATGTCAGTTCCGTTAAGAATGCTGAGGACATTACATCACACGTTCCTAACTACATCCCTAATGGTGTGTTCAGTATTTGCGGAAGTGGTACGGAAAACTTCTGTTCGGTACTATCTCACGGGGACCCTAGTAAAATCTTCATGt                                >  1:13167/1‑301 (MQ=255)`  
                                                                                                                                                                                                                                                                                             `aTCTAAGTCGGTTGCGTTGAACCTAACGACCCAGTTTGACGTACAGGACCGAGCGAGACCTTTTGGGATTGGGCGTAATGTCTACTTTGCTAGTCCGAGGTCCAGCTTCACGTCCATCCACAGGTACTACGCTGTGCAGGATGTCAGTTCCGTTAAGAATGCTGAGGACATTACATCACACGTTCCTAACTACATCCCTAATGGTGTGTTCAGTATTTGCGGAAGTGGTACGGAAAACTTCTGTTCGGTACTATCTCACGGGGACCCTAGTAAAATCTTCATGTACAAATTCCTGTACCtt               >  1:492695/1‑300 (MQ=255)`  
                                                                                                                                                                                                                                                                                                `tAAGTCGGTTGAGTTGAACCTAACGACCCAGTTTGACGTACAGGACCGAGCGAGACCTTTTGGGATTGGGCGTAATGTCTACTTTGCTAGTCCGAGGTCCAGCTTCACGTCCATCCACAGGTACTACGCTGTGCAGGATGTCAGTTCCGTTAAGAATGCTGAGGGCATTACATCACACGTTCCTAACTACATCCCTAATGGTGTGTTCAGTATTTGCGGAAGTGGTACGGAAAACTTCTGTTCGGTACTATCTCACGGGGACACTAGTAAAATCTTCATGTACAAATTCCTGTACCTGAAc            >  1:100120/1‑301 (MQ=255)`  
                                                                                                                                                                                                                                                                                                  `agcaggTTGGATTTAACCTAACGACCCAGTTTGACGTACAGGACCGAGCGAGACCTTTTGGGATTGGGCGTAATGTCTACTTTGCTAGTCCGAGGTCCAGCTTCACGTCCATCCACAGGTACTACGCTGTGCAGGATGTCAGTTCCGTTAAGAATGCTGAGGACATTACATCACACGTTCCTAACTACATCCCTAATGGTGTGTTCAGTATTTGCGGAAGTGGTACGGAAAACTTCTGTTCGGTACTATCTCACGGGGACCCTAGTAAAATCTTCATGTACAAATTCCTGTACCTGAACGa          <  1:371862/297‑1 (MQ=255)`  
                                                                                                                                                                                                                                                                                                   `gTCGGTTGGGTTGAACCTAACGACCCAGTTTGACGTACAGGACCGAGCGAGACCTTTTGGAATTGGGCGTAATGTCTACTTTGCTAGTCCGAGGTCCAGGTTCACATCCATCCACAGGTACTACGCTGTGCAGGATGTCAGTTCCGTTAAGAATGCTGAGGACATTACATCACACGTTCCTAACTACATCCCTAATGGTGTGTTCAGTATTTGCGGAAGTGGTACGGAAAACTTCTGTTCGGTACTATCTCACGGGGATCATAGTAAAATCTTCATGTACAAATTACTGTACCTGAACGaa         >  1:1413/1‑301 (MQ=255)`  

                                                                                                                                                                                                                                                                                                           `|`                                                                                                                                                                                                                                                                                                             
`CACCAACCCTTGGCCTTCTTTTGTTGGTTCAAGTATTAACGATGTGTTCTTCTTCCGTAACCGCTTAGGATTCCTTAGTGGGGAGAACATCATATTGAGTCGTACAGCCAAATACTTCAACTTCTACCCTGCGTCCATTGCGAACCTTAGTGATGACGACCCTATAGACGTAGCTGTGAGTACCAACCGAATAGCAATCCTTAAGTACGCCGTTCCGTTCTCAGAAGAGTTACTCATCTGGTCCGATGAAGCACAATTCGTCCTGACTGCCTCGGGTACTCTCACATCTAAGTCGGTTGAGTTGAACCTAACGACCCAGTTTGACGTACAGGACCGAGCGAGACCTTTTGGGATTGGGCGTAATGTCTACTTTGCTAGTCCGAGGTCCAGCTTCACGTCCATCCACAGGTACTACGCTGTGCAGGATGTCAGTTCCGTTAAGAATGCTGAGGACATTACATCACACGTTCCTAACTACATCCCTAATGGTGTGTTCAGTATTTGCGGAAGTGGTACGGAAAACTTCTGTTCGGTACTATCTCACGGGGACCCTAGTAAAATCTTCATGTACAAATTCCTGTACCTGAACGAAGAGTTAA  >  NC_001604.1/25849‑26447`

  

Alignment Legend

Aligned base mismatch/match (shaded by quality score): `ATCG/ATCG` `< 3 ≤` `ATCG/ATCG` `< 10 ≤` `ATCG/ATCG` `< 21 ≤` `ATCG/ATCG` `< 34 ≤` `ATCG/ATCG` `< 38 ≤` `ATCG/ATCG`

Unaligned base: `atcg`    Masked matching base: `atcg`    Alignment gap: `‑`    Deleted base: `‑`
